# Mortality trends for chronic kidney disease and dementia in the United States, 1999–2020: a 22-year retrospective analysis

**DOI:** 10.1097/MS9.0000000000004761

**Published:** 2026-01-20

**Authors:** Muhammad S. Ahmad, Sarmad Imran, Muhammad A. Kamal, Archi Mehta, Iqra Nawaz, Iman O. Abufatima, Abdulhadi M.A. Mahgoub, Mohammedsadeq A. Shweliya

**Affiliations:** a Department of Medicine, CMH Lahore Medical College, Lahore, Pakistan; b Department of Medicine, Quaid-e-Azam Medical College Bahawalpur, Bahawalpur, Pakistan; c Department of Medicine, GMERS Medical College Gotri, Vadodara, Gujarat, India; d Department of Medicine, University of Medical Sciences and Technology, Khartoum, Sudan; e Department of Medicine, University of Gezira, Wad-Medani, Gezira, Sudan; f Department of Medicine, University of Baghdad College of Medicine, Baghdad, Iraq

**Keywords:** CDC WONDER, chronic kidney disease, dementia, mortality trends

## Abstract

**Background:**

Chronic kidney disease (CKD) and dementia are major causes of morbidity and mortality among older adults and frequently coexist due to shared vascular and metabolic risk factors. Despite their clinical importance, national mortality trends for these conditions remain underexplored. This study aimed to examine temporal and demographic patterns of mortality among individuals with coexisting CKD and dementia in the United States from 1999 to 2020.

**Methods:**

In this retrospective, population-based study, mortality data for adults aged ≥25 years were extracted from the Centers for Disease Control and Prevention’s Wide-ranging Online Data for Epidemiologic Research database. Deaths listing both CKD [International Statistical Classification of Diseases and Related Health Problems, 10th Revision (ICD-10 N18.x)] and dementia (ICD-10 F01.x, F03, G30.x) as causes were included. Age-adjusted mortality rates (AAMRs) per 100 000 were calculated using the 2000 U.S. standard population. Temporal trends were analyzed using Joinpoint regression to estimate annual percent change and average annual percent change (AAPC) with *P* < 0.05 considered significant. Subgroup analyses were stratified by gender, race/ethnicity, age, region, and urbanization.

**Results:**

Between 1999 and 2020, 170 375 deaths were attributed to CKD and dementia. The overall AAMR increased from 1.03 to 5.92 per 100 000 (AAPC 7.41%). Mortality increased significantly across both genders, older adults, and all census regions, peaking in 2012, briefly declining between 2012 and 2015, then rising during 2015 to 2020. Males had higher mortality (AAMR 4.24) than females (3.16). Non-Hispanic Black individuals exhibited the highest rate (6.20), followed by non-Hispanic White (3.35) and Hispanic (3.09) populations. The Midwest showed the highest regional burden (AAMR 3.90), while rural counties recorded greater mortality (3.89) than urban areas (3.49).

**Conclusion:**

The increasing mortality trends show a close relationship between CKD and dementia. Thus, improving early diagnosis, ensuring equal access to care, and providing a holistic treatment approach targeting both the organs is key to enhancing optimal patient care and consequently reducing mortality.

## Background

With estimated prevalence rate of 11–13%, chronic kidney disease (CKD) continues to contribute significantly to global health burden, particularly among the ageing population^[^1^]^. CKD is characterized by a reduction in estimated glomerular filtration rates (eGFR) of <60 mL/min/1.73 m^2^ or by elevated levels of albuminuria, ≥30 mg of albumin per 1 g of creatinine, lasting for a duration of 3 months or more^[^2^]^. Among other complications associated with CKD, neurological complications, including cognitive dysfunction and dementia, represent an important subset of clinical manifestations observed with renal dysfunction. This assumes greater importance as CKD serves as an independent contributing factor leading to cognitive impairment and dementia^[^3^]^. Dementia, which refers to a progressive multi-domain deterioration of cognition and social and/or occupational functioning exceeding that expected of the normal aging, is linked to higher rates of morbidity and death^[^4^]^.

**HIGHLIGHTS**

- This 21-year analysis examined mortality trends from chronic kidney disease (CKD) and dementia in the U.S. using Centers for Disease Control and Prevention’s Wide-ranging Online Data for Epidemiologic Research data (1999–2020).
- A total of 170 375 deaths were reported to coexisting CKD and dementia.
- Mortality rate was higher among males, non-Hispanic Black individuals, and rural residents.
- The age-adjusted mortality rate increased from 1.03 to 5.92 per 100 000 (average annual percent change 7.41%), representing nearly sixfold rise.
- Mortality increased significantly across both genders, older adults, and all census regions, peaking around 2012, followed by a brief decline from 2012 to 2015, then rising again through 2020.

Previously published epidemiological studies, retrieved through public databases including PubMed, MEDLINE, and PubMed Central using a comprehensive search strategy of relevant keywords while following a strict inclusion criteria, have demonstrated that patients at every stage of CKD may be exposed to an increased risk of dementia as well as other cognitive disorders^[^1^]^. Furthermore, a 10 mL/min/1.73 m^2^ reduction in eGFR was reported to be associated with an 11% higher prevalence of cognitive dysfunction^[^5^]^. Besides impaired baseline kidney function, a more rapid deterioration is linked to an increased likelihood of dementia development^[^6^]^. In contrast to the general population, a three- to fivefold rise in burden of dementia is reported in patients undergoing dialysis^[^7^]^.

Various genetic biomarkers have been described in association with CKD and cognitive dysfunction, demonstrating a shared genetic background between the two^[^8^]^. The pathogenesis of CKD leading to impaired cognition has been attributed to both vascular and neurodegenerative processes^[^9^]^. Similarities in anatomical structures and physiological features shared by kidney and the brain render both these organs particularly sensitive to the effects of traditional risk factors including hypertension, smoking, and diabetes mellitus^[^1^]^. Moreover, risk factors specific to CKD including anemia and electrolyte disturbances such as hyponatremia have been associated with dementia^[^10^]^. Furthermore, oxidative stress, chronic inflammation, accumulation of uremic toxins, and increased levels of cystatin-C all contribute to impaired cognition^[^3^]^. With a prominent overlap in pathophysiological mechanisms underlying CKD and dementia, it becomes challenging to distinguish causal associations from mediators, confounders, and coincidental associations.Cognitive impairment remains underdiagnosed despite being recognized as a frequently occurring complication. Published data report that 30–60% of patients on dialysis exhibit cognitive impairment, yet fewer than 5% have a formally documented clinical diagnosis^[^11^]^. Furthermore, dementia serves as a strong predictor of mortality among dialysis patients^[^11,12^]^. Therefore, we examine mortality trends among patients with CKD and dementia through a retrospective analysis using mortality data from Centers for Disease Control and Prevention’s Wide-ranging Online Data for Epidemiologic Research (CDC WONDER) database from 1999 to 2020, and stratify results in terms of gender, age, region, race, and setting in order to identify underlying health disparities.

## Methods

### Study design

We conducted a retrospective, population-based study using the CDC WONDER Multiple Cause-of-Death (MCD) Public Use database for calendar years 1999–2020^[^13^]^. The analytic cohort comprised adults aged ≥25 years. Records were eligible if both dementia and CKD were recorded anywhere on the death certificate (underlying or contributing causes) in the MCD file (which includes up to 20 contributing causes)^[^13^]^. We employed the International Statistical Classification of Diseases and Related Health Problems, 10th Revision (ICD-10) coding system to perform diagnostic coding where dementia was identified using ICD-10 codes F01.0–F01.9, F03, and G30.0–G30.9; CKD was identified using ICD-10 codes N18.0–N18.5, N18.8, and N18.9^[^14^]^. Because CDC WONDER contains de-identified public-use data, institutional review board approval was not required. Reporting followed the STROBE recommendations for observational studies^[^15^]^.

### Data abstraction

From CDC WONDER, two independent authors extracted annual death counts and population estimates along with gender, race/ethnicity, U.S. Census region, National Center for Health Statistics (NCHS) urban-rural county classification, 10-year age groups, and place of death^[^13^]^. Race and ethnicity were grouped as: non-Hispanic (NH) White, NH Black or African American, Hispanic or Latino, NH American Indian or Alaska Native (AI/AN), and NH Asian or Pacific Islander (API). Geographic regions (Northeast, Midwest, South, and West) followed U.S. Census Bureau definitions^[^16^]^. Urbanization used the 2013 NCHS Urban–Rural Classification Scheme for Counties, grouped as large metropolitan areas (population ≥1 000 000), medium/small metropolitan areas (50 000–999 999), and rural areas (<50 000)^[^17^]^. Place of death was abstracted as defined in CDC WONDER and categorized as inpatient hospital, outpatient/ER, dead on arrival, home, hospice facility, nursing home/long-term care, and other/unknown.

For all deaths meeting inclusion criteria, we computed age-adjusted mortality rates (AAMRs) per 100 000 population using the direct method and the year 2000 U.S. standard population; rates were summarized overall and stratified by gender, race/ethnicity, region, urbanization category, place of death, and 10-year age group^[^18^]^. Values suppressed by WONDER for confidentiality or stability, or flagged as statistically unreliable, were not analyzed further^[^13,19^]^.

### Statistical analysis

Temporal trends in AAMRs from 1999 to 2020 were assessed using Joinpoint regression (Joinpoint Regression Program, version 5.1.0.0; National Cancer Institute)^[^20^]^. Log-linear models were fit to annual AAMRs to estimate annual percent change (APC) for each segment and average annual percent change (AAPC) across the full interval, with 95% confidence intervals (CIs)^[^20–22^]^. The number and timing of joinpoints were determined via Monte Carlo permutation testing at an overall α = 0.05, per program defaults^[^21^]^. The minimum and maximum number of joinpoints used was according to the guidelines which were 0–4. A two-tailed *P* < 0.05 denoted statistical significance for APC/AAPC estimates^[^20–22^]^. Trend analyses were conducted overall and stratified by gender, race/ethnicity, and census region.

To preserve statistical reliability, subgroup–year estimates based on <20 deaths or with relative standard error ≥23% were excluded from trend modeling, consistent with NCHS/CDC guidance^[^19^]^. Accordingly, NH AI/AN and 10-year age groups 25–34, 35–44, and 45–54 were excluded from Joinpoint analyses due to sparse counts and unstable rates but were retained in descriptive AAMR summaries when estimates were reportable^[^19^]^. All calculations used aggregated outputs from CDC WONDER^[^13^]^.

## Results

**Figure 1.**
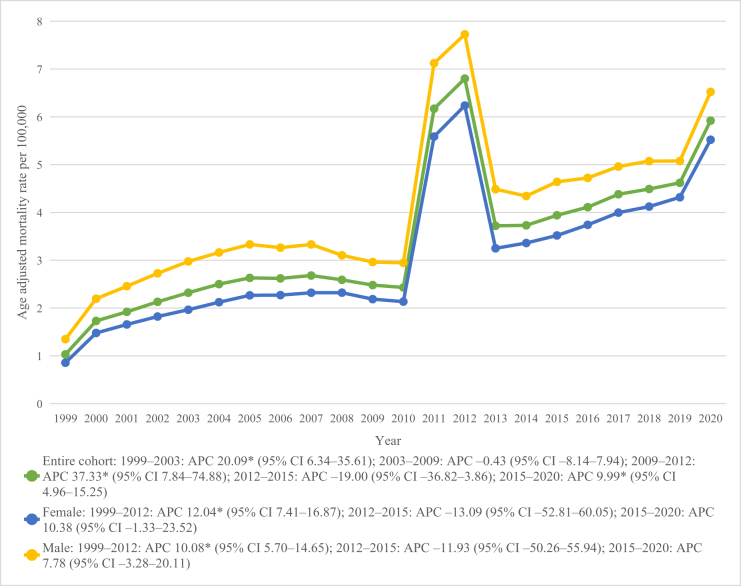
Overall and gender-wise chronic kidney disease and dementia mortality trends in the United States from 1999 to 2020.

A total of 170 375 deaths were reported due to CKD and dementia between 1999 and 2020 (Table 1). Overall AAMR was 3.56 (95% CI 3.54–3.57), increasing from 1.03 in 1999 to 5.92 in 2020 with an AAPC of 7.41% (2.04–13.07; Fig. 1). Mortality increased significantly from 1999 to 2003 at 20.09% per year, remained stable during 2003–2009, and surged again between 2009 and 2012 at 37.33% per year (Table 1; Fig. 1). A decline was noted from 2012 to 2015 though not statistically significant, followed by a renewed and significant increase from 2015 to 2020 (APC = 9.99%; 95% CI 4.96–15.25; Table 2; Fig. 1).

**Table 1:** Demographic characteristics of chronic kidney disease and dementia-related mortality in the United States from 1999 to 2020

Variable	Deaths	Population	AAMR (95% CI)
Overall			
1999–2020	170375	4473854489	3.56 (3.54–3.57)
Gender			
Male	74115	2154556911	4.24 (4.21–4.27)
Female	96260	2319297578	3.16 (3.14–3.18)
Age group^a^			
55–64 years	2108	766424847	0.28 (0.26–0.29)
65–74 years	11370	510458341	2.23 (2.19–2.27)
75–84 years	48160	298504433	16.13 (15.99–16.28)
85+ years	108412	119513891	90.71 (90.17–91.25)
Race/ethnicity^b^			
NH American Indian or Alaska Native	660	33 081 153	3.44 (3.17–3.71)
NH Asian or Pacific Islander	3929	237142712	2.54 (2.46–2.62)
NH Black or African American	23659	518937524	6.33 (6.25–6.41)
NH White	132875	3095342890	3.58 (3.57–3.60)
Hispanic or Latino	8937	589 350 210	3.09 (3.02–3.15)
Census region			
Northeast	27810	827193779	2.78 (2.75–2.82)
Midwest	43498	969567311	3.90 (3.86–3.93)
South	62059	1652256217	3.71 (3.68–3.73)
West	37008	1024837182	3.69 (3.65–3.72)
Urbanization			
Urban	137190	3795213822	3.49 (3.47–3.51)
Rural	33185	678634169	3.89 (3.84–3.93)
Place of death			
Medical facility	38608		
Decedent’s home	28692		
Hospice facility	8586		
Nursing home/long-term care	85 201		
Other	9021		
Place of death unknown	267		

AAMR, age-adjusted mortality rate; CI, confidence interval; NH, non-Hispanic.

^a^ Crude mortality rate is used for analysis instead of age-adjusted mortality rates for age groups.

^b^ Few cases were not stated.

**Table 2:** APCs and AAPCs in CKD- and dementia-related mortality in the United States from 1999 to 2020

Variable	Trend segment	Lower endpoint	Upper endpoint	APC (95 % CI)	AAPC	*P* value
Entire cohort	1	1999	2003	20.09* (6.34–35.61)	7.41* (2.04–13.07)	0.006
	2	2003	2009	−0.43 (−8.14–7.94)		
	3	2009	2012	37.33* (7.84–74.88)		
	4	2012	2015	−19.00 (−36.82–3.86)		
	5	2015	2020	9.99* (4.96–15.25)		
Gender						
Female	1	1999	2012	12.04* (7.41–16.87)	7.67 (−1.27–17.42)	0.094
	2	2012	2015	−13.09 (−52.81–60.05)		
	3	2015	2020	10.38 (−1.33–23.52)		
Male	1	1999	2012	10.08* (5.70–14.65)	6.10 (−2.21–15.11)	0.155
	2	2012	2015	−11.93 (−50.26–55.94)		
	3	2015	2020	7.78 (−3.28–20.11)		
Age group						
55–64 years	1	1999	2012	2.96 (−0.56–6.61)	0.76 (−2.15–3.75)	0.613
	2	2012	2020	−2.73 (−8.42–3.31)		
65–74 years	1	1999	2011	5.54* (0.75–10.55)	2.25 (−0.86–5.45)	0.157
	2	2011	2020	−1.97 (−6.44–2.70)		
75–84 years	1	1999	2020	4.36* (2.05–6.72)	4.36* (2.05–6.72)	<0.001
85+ years	1	1999	2020	6.32* (3.92–8.77)	6.32* (3.92–8.77)	<0.001
Race/ethnicity^1^						
NH Asian or Pacific Islander	1	1999	2003	26.60 (–3.65–66.33)	5.03 (–4.29–15.42)	0.308
	2	2003	2008	–8.29 (–22.57–8.61)		
	3	2008	2011	47.86 (–12.21–149.06)		
	4	2011	2014	–22.49 (–44.18–7.64)		
	5	2014	2020	1.85 (–3.58–7.59)		
NH Black or African American	1	1999	2002	24.55 (–2.33–58.68)	4.63 (–2.09–11.80)	0.181
	2	2002	2009	–1.96 (–7.96–4.42)		
	3	2009	2012	31.56 (–4.79–81.80)		
	4	2012	2015	−23.50 (−44.51–5.45)		
	5	2015	2020	8.56* (2.15–15.38)		
NH White	1	1999	2003	20.02* (6.74–34.96)	8.29* (2.82–14.06)	0.003
	2	2003	2009	1.02 (–6.36–8.98)		
	3	2009	2012	35.31* (1.57–80.27)		
	4	2012	2015	−17.51 (−33.59–2.47)		
	5	2015	2020	11.68* (6.75–16.83)		
Hispanic or Latino	1	1999	2003	18.77* (4.35–35.17)	6.62* (2.15–11.29)	0.003
	2	2003	2009	2.32 (−3.66–8.67)		
	3	2009	2012	35.27* (11.63–63.92)		
	4	2012	2015	−25.69* (−39.17–−9.21)		
	5	2015	2020	10.65* (6.94–14.48)		
Census region						
Northeast	1	1999	2001	44.53 (−6.16–122.59)	8.77* (2.80–15.08)	0.004
	2	2001	2009	2.20 (−2.10–6.70)		
	3	2009	2012	33.01* (4.48–69.31)		
	4	2012	2015	−16.21 (−33.93–6.26)		
	5	2015	2020	11.15* (6.46–16.04)		
Midwest	1	1999	2003	20.86* (3.10–41.68)	8.05* (1.70–14.79)	0.012
	2	2003	2009	−0.26 (−8.23–8.84)		
	3	2009	2012	36.67* (2.11–82.93)		
	4	2012	2015	−17.98 (−38.59–9.61)		
	5	2015	2020	11.41* (5.93–17.17)		
South	1	1999	2002	21.04* (0.98–45.07)	6.43* (1.84–11.24)	0.006
	2	2002	2009	1.01 (−3.14–5.34)		
	3	2009	2012	33.89* (8.57–65.11)		
	4	2012	2015	−19.94* (−34.64 to −1.93)		
	5	2015	2020	9.57* (5.39–13.92)		
West	1	1999	2003	24.23* (6.00–45.60)	8.24* (2.65–14.13)	0.003
	2	2003	2009	0.34 (−6.60–7.78)		
	3	2009	2012	41.41* (10.43–81.08)		
	4	2012	2015	−20.57 (−37.65–1.19)		
	5	2015	2020	8.89* (4.22–13.77)		
Urbanization						
Urban	1	1999	2002	23.52 (−4.84–60.34)	7.24* (1.12–13.74)	0.020
	2	2002	2009	1.32 (−4.45–7.44)		
	3	2009	2012	34.90* (0.18–81.65)		
	4	2012	2015	−18.78 (−35.54–2.35)		
	5	2015	2020	9.83* (5.01–14.88)		
Rural	1	1999	2004	15.21* (6.28–24.90)	7.55* (2.77–12.55)	0.002
	2	2004	2009	0.85 (−7.41–9.48)		
	3	2009	2012	30.52* (4.12–63.62)		
	4	2012	2015	−15.83 (−32.15–4.42)		
	5	2015	2020	10.41* (6.16–14.83)		

APC, annual percent change; AAPC, average annual percent change; CKD, chronic kidney disease; CI, confidence interval; NH, non-Hispanic. *P-value less than 0.05.

^1^ Trend analysis was not possible for NH American Indian or Alaska Natives; there was unreliable data for few years.

### Trends by gender

Males had a higher overall AAMR of 4.24 compared to females, who had an overall AAMR of 3.16 (Table 1). Both genders followed a similar pattern, with a significant rise from 1999 to 2012 (female APC: 12.04; male APC: 10.08), followed by a non-significant decline between 2012 and 2015 and a non-significant upward trend from 2015 to 2020. However, the overall mortality trend was not statistically significant, with an AAPC of 6.10% in males and 7.67% in females (Table 2; Fig. 1).

### Trends by race/ethnicity

**Figure 2.**
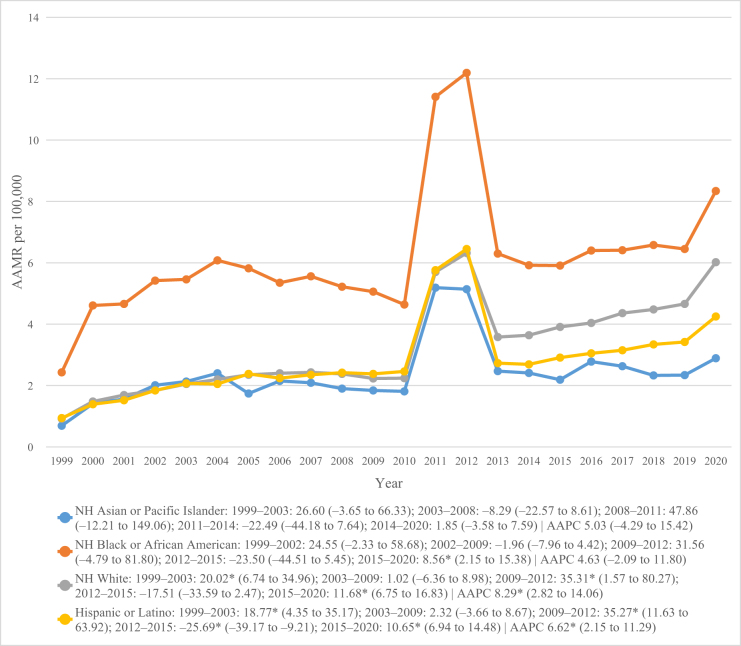
Chronic kidney disease and dementia-related mortality trends stratified by race/ethnicity in the United States from 1999 to 2020.

Across racial and ethnic groups, AAMR was highest among NH Black individuals (AAMR = 6.33; 95% CI 6.12–6.41), followed by NH White (3.58; 3.57–3.60), NH AI/AN (3.44; 3.17–3.71), Hispanic or Latino (3.09; 95% CI 3.02–3.15), and NH API populations (2.54; 95% CI 2.46–2.62; Table 1). All groups displayed a similar mortality rate pattern – an early rise until 2011–2012, a brief decline around 2012–2015, and a subsequent upward trend after 2015 (Fig. 2). Notably, NH White (AAPC = 8.29%) and Hispanic or Latino populations (AAPC = 6.62%) demonstrated statistically significant overall changes across the study period, driven by distinct surges during 1999–2003 (NH White APC = 20.02%; Hispanic APC = 18.77%) and 2009–2012 (NH White APC = 35.31%; Hispanic APC = 35.27%). This was followed by a non-significant decline among NH Whites and a significant drop among Hispanics (APC = −25.69%) during 2012–2015, with both groups showing significant mortality rebounds between 2015 and 2020. In contrast, NH Black and NH API groups exhibited smaller, non-significant fluctuations in mortality (AAPC = 4.55% and 5.08%, respectively; Table 2; Fig. 2).

### Trends by census region and states

**Figure 3.**
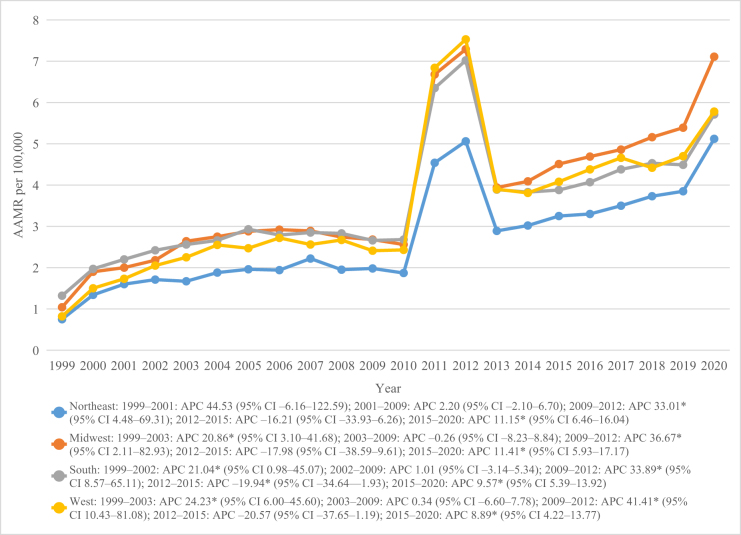
Chronic kidney disease and dementia-related mortality trends stratified by census region in the United States from 1999 to 2020.

Across census regions, the Midwest had the highest overall mortality rate (AAMR = 3.90; 95% CI 3.86–3.93), followed by the South (AAMR = 3.71; 3.68–3.73), West (AAMR = 3.69; 3.65–3.72), and Northeast (AAMR = 2.78; 2.75–2.82; Table 1). Significant increases were noted across all regions during 2009–2012 and 2015–2020, while the mid-decade declines (2012–2015) were generally non-significant, except for the South, where the decrease was significant (APC = −19.94; 95% CI −34.64 to −1.93). Overall, all four regions exhibited significant long-term increases in mortality, with the Northeast showing the steepest upward trend (AAPC = 8.76; 95% CI 2.80–15.08), followed closely by the West (AAPC = 8.24; 95% CI 2.65–14.13; Table 2; Fig. 3). States with the highest overall AAMR included Colorado (5.74), Minnesota (5.70), and South Carolina (5.33), while the lowest rates occurred in Nevada (1.68), Arizona (2.03), and Florida (2.04).

### Trends by urbanization

**Figure 4.**
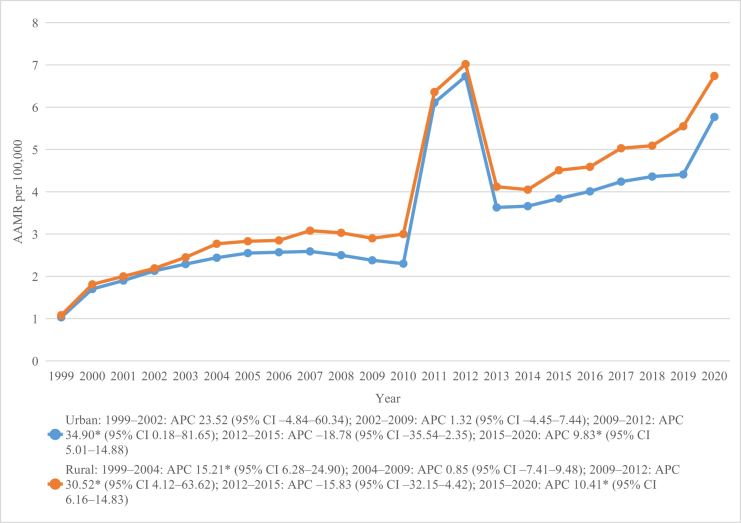
Chronic kidney disease and dementia-related mortality trends stratified by 2013 urbanization in the United States from 1999 to 2020.

Non-metropolitan regions had higher mortality (AAMR = 3.89; 95% CI 3.84–3.93) than metropolitan areas (AAMR = 3.49; 95% CI 3.47–3.51; Table 1). Both showed significant increases during 2009–2012 (metropolitan APC = 34.90%; non-metropolitan APC = 30.52%) and 2015–2020 (metropolitan APC = 9.83%; non-metropolitan APC = 10.41%), while mid-decade declines (2012–2015) were non-significant. Overall, long-term mortality rise significantly in both settings (metropolitan AAPC = 7.24%; non-metropolitan AAPC = 7.55%; Table 2; Fig. 4).

### Trends by 10-year age groups

**Figure 5.**
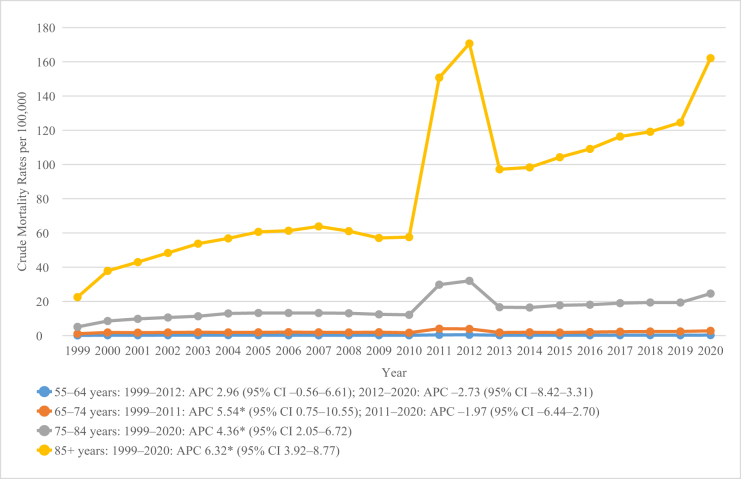
Chronic kidney disease and dementia-related mortality trends stratified by 10-year age groups in the United States from 1999 to 2020.

From 1999 to 2020, mortality among the 55–64 year age group showed no significant change (AAPC = 0.76; 95% CI −2.15–3.75). The 65–74 year age group increased significantly from 1999–2011 (APC = 5.54; 95% CI 0.75–10.55) before a non-significant decline (AAPC = 2.25; 95% CI −0.86–5.45). Mortality rose significantly in older groups – 75–84 years (AAPC = 4.35; 95% CI 2.05–6.72) and 85+ years (AAPC = 6.32; 95% CI 3.92–8.77), with the oldest group peaking around 2012, briefly dipping, then rising again toward 2020 (Table 2; Fig. 5).

### Distribution by place of death

Out of the total 170 375 deaths over the 21 years, most deaths occurred in nursing homes or long-term care facilities (50.0%), followed by medical facilities (22.7%) predominantly among inpatients (20.6%), with fewer at home (16.8%) and hospice centers (5.0%; Table 1).

## Discussion

Findings from our analysis of national mortality trends associated with CKD and dementia report a total of 170 375 deaths with an overall AAMR of 3.56. In terms of gender, males had higher mortality (AAMR = 4.24) as compared to the female population (AAMR = 3.16). In context of race, NH Black individuals exhibited the highest racial mortality burden (AAMR = 6.20). Regionally, Midwest showed the highest mortality rate (AAMR = 3.90) and with a greater mortality rate observed in the non-metropolitan areas (AAMR = 3.89) compared to the metropolitan settings (AAMR = 3.49). Older adults, particularly those 85 years or older, had the steepest increase. Additionally, half of all deaths occurred in nursing homes or long-term care facilities.

A detailed literature search of PubMed/MEDLINE/PubMed Central was conducted using relevant keywords, alone and in combination with various Boolean operators to yield relevant evidence. Studies retrieved from our search strategy highlight that the coexistence of CKD and dementia contributes to elevated mortality through overlapping and synergistic pathophysiological mechanisms. CKD promotes systemic vascular dysfunction including hypertension, diabetes related microvascular injury, and atherosclerosis, which impairs the cerebral perfusion and increases the risk of cerebrovascular events, such as microinfarcts and white-matter lesions thereby directly contributing to the cognitive decline^[^3^]^. Chronic inflammation and oxidative stress associated with CKD exacerbate the neuronal injury, while accumulation of uremic toxins including gut-derived neurotoxins promotes neurotoxicity and further accelerates neurodegenerative processes^[^23,24^]^. Reduced eGFR and albuminuria independently predict cognitive impairment which highlight that renal dysfunction is a key driver for dementia progression^[^25,26^]^. Additionally, CKD-related metabolic disturbances, anemia, and impaired drug clearance increase the vulnerability to acute illnesses, hospitalizations, and complications, thereby further increasing the mortality burden^[^8^]^. Together, these interconnected vascular, inflammatory, and neurotoxic processes create a vicious cycle in which CKD accelerates the cognitive decline, while dementia increases the vulnerability to CKD-related complications which ultimately contribute to substantially higher mortality observed in individuals with both conditions^[^27^]^.

The overall AAMR increased sharply from 1.03 in the year 1999 to 5.92 in 2020, with a significant surge during 1999–2003 and 2009–2012, followed by a brief decline and a rebound after 2015. These trends in our analysis closely mirror broader, nationally representative mortality patterns for dementia^[^28^]^. Marked fluctuations in these pattern highlight the periods of rising risk, stabilization, and resurgence, emphasizing the need for the early detection, integrated healthcare, and targeted public health strategies to reduce mortality burden from these coexisting conditions.

Between year 1999 and 2020, the overall AAMR for the coexisting CKD and dementia increased significantly with males exhibiting a higher AAMR (4.24) compared to females (3.16). Both genders followed a similar pattern with a rise in AAMR from 1999 to 2012, followed by a short decline and a subsequent increase from 2015 to 2020. Male predominance in CKD-related mortality, as observed in our study, is consistent with findings from previous large-scale studies specifically, a Global Burden of Disease study which demonstrated higher mortality rates among males globally. Additionally, results from a recent clinical trial as well as a National Institutes of Health based cohort reported similar male biased trends in the U.S., thus supporting our results^[^29–31^]^. In contrast, population level epidemiologic analysis using national registry data by Shoaib *et al* found that dementia-related mortality was higher among females than males^[^28^]^. The observed gender difference is likely shaped by multiple interrelated social and gender factors, such as variations in access to medical care, kidney replacement therapy, and therapies aimed at slowing disease progression^[^32,33^]^. Moreover, gender-specific behavioral factors such as the financial hardship and lower engagement in cognitive and physical activities in men further contribute to the divergent dementia risk trajectories and mortality outcomes^[^34^]^. Future strategies should be tailored and focused on addressing these gender related disparities through equitable access to the healthcare, early risk assessment, and targeted interventions to reduce the higher CKD and dementia associated mortality observed in men.

Mortality associated with coexisting CKD and dementia was increased markedly with the age, remaining stable in the 55–64 group but rising significantly in those aged ≥65 and peaking in the oldest adults. Our findings align with the large, population-based U.S. cohorts, which similarly demonstrate that higher variability in eGFR, advanced CKD, and hemodialysis in patients with dementia is associated with elevated risks of cognitive decline and mortality^[^35–37^]^. Addressing mortality in the older adults with CKD and dementia requires close monitoring for cognitive decline, proactive management for the vascular and metabolic risk factors, and integrated multidisciplinary care to decrease the mortality burden.

Our study reported that the NH Black adults had the highest mortality from the coexisting CKD and dementia, followed by NH White, Hispanic, and NH API populations with all the groups showing similar temporal trends of early rise then brief decline and later on increases, reflecting the persistent racial disparities. These findings are consistent with earlier research using data from the Centers for Medicare and Medicaid Services Medicare Beneficiary Summary File, which has consistently reported notable racial and ethnic disparities in dementia- and CKD-related mortality, with NH Black individuals experiencing disproportionately higher rates^[^38,39^]^. The higher dementia and CKD-related mortality among NH Black individuals maybe primarily driven by the greater prevalence of disease, delayed or missed diagnoses, limited healthcare access and utilization, socioeconomic disadvantages, structural racism, and potential genetic or biological factors^[^40–42^]^. These results emphasize the critical need for the targeted interventions, health equity initiatives, and systemic reforms to address these persistent racial disparities in dementia and CKD-related mortality.

Regional disparities were very evident in CKD- and dementia-related mortality in the U.S. with the Midwest showing the highest overall AAMR followed by the South, West, and Northeast, and all regions demonstrating the significant long term increases particularly in the region of Northeast and West. Although research on the regional variation in CKD and dementia related mortality is limited, prior studies have consistently reported a higher all-cause mortality in the Southern U.S. compared to the other regions^[^43–45^]^. It is not very well understood why mortality is elevated in the Midwestern U.S.; however, the potential contributors might include the limited healthcare access, lower quality of care, socioeconomic disadvantages, unhealthy lifestyle behaviors, environmental exposures, and regional variations in the healthcare infrastructure. By addressing these disparities, public health interventions and further research needed to elucidate these region-specific determinants.

Non-metropolitan regions demonstrated higher long-term mortality rates compared to metropolitan areas. Our findings are consistent with the growing body of cohort and population level evidence that show that rural and non-metropolitan residents experience higher mortality burden and worse care trajectories for both CKD and dementia compared with the urban residents^[^46–48^]^. The persistently higher mortality rates among the rural populations in the U.S. can be attributed to a combination of various factors including limited access to the healthcare and specialists, socioeconomic deprivation, higher prevalence of the modifiable risk factors, hospital closures, and an aging population with fewer caregiving resources^[^49–53^]^. Future efforts should prioritize enhancing access to healthcare delivery, specialist availability, and preventive programs in rural regions through telemedicine, and a stronger workforce to reduce the persistent mortality gap.

The majority of the deaths occurred in the nursing homes or long-term care facilities, followed by the inpatient deaths in medical facilities with fewer occurring at home or hospice settings indicating that end of life care for chronic and progressive conditions such as CKD and dementia remains predominantly institutionalized. Prior studies have also showed high proportions of nursing home deaths among dementia patients^[^54^]^. The relatively lower proportion of home and hospice deaths highlights the potential gaps in the community based palliative care, caregiver support, and timely access to hospice services, emphasizing the need to strengthen the non-institutional end of life care options for the patients with complex chronic diseases.

This study utilized the nationally representative CDC data over two decades to provide a robust, population level analysis of the coexisting CKD and dementia, offering insights to guide healthcare planning and resource allocation. These findings underscore the need for the targeted interventions such as expanding telemedicine services, promoting equitable access to preventive care, to reduce disparities and to improve outcomes in the vulnerable populations.

## Future directions

Mounting evidence indicates that the association of CKD and dementia represents a biologically linked, high-fatality syndrome rather than the coincidental comorbidity. Future guidelines should reconceptualize the convergence of CKD and dementia as a priority multimorbidity requiring early, coordinated prevention rather than late-stage management. Operationalizing the kidney-brain axis framework offers a pragmatic pathway for midlife identification of the high-risk trajectories through integrated risk stratification, biomarker informed screening, and longitudinal population surveillance. Future directions must also prioritize the prevention-focused trials, cognition-sensitive treatment pathways, and anticipatory care models embedded within the chronic disease programs. Collectively, such a shift has the potential to decline otherwise predictable mortality trajectories and meaningfully redefine the outcomes for high risk populations.

## Limitations

There are certain limitations that cannot be overlooked. First, this study relies on data obtained from the death certificate which may be the subject to misclassification or underreporting of underlying conditions such as CKD and dementia. Second, the dataset does not have detailed information about comorbidities, disease severity, treatment patterns, and socioeconomic factors at the individual level which limits the ability to fully explain the observed disparities. Finally, the temporal trends and regional differences which may be influenced by the changes in coding practices, diagnostic criteria, and variations in healthcare infrastructure or access could affect the comparability of mortality rates across years and settings.

## Conclusion

This study provides nationally representative, long-term evidence showing rising mortality in coexisting CKD and dementia, with the male population, NH Black adults, older individuals, and rural residents disproportionately affected. These findings inform public health policy, guiding targeted interventions, equitable resource allocation, and the expansion of the community and home-based care programs to reduce preventable deaths and address persistent demographic, racial, and geographic disparities.

## Data Availability

The datasets generated and analyzed during the current study are available in the CDC WONDER database, https://wonder.cdc.gov.
